# Insight into the Molecular Weight of Hydrophobic Starch Laurate-Based Adhesives for Paper

**DOI:** 10.3390/polym15071754

**Published:** 2023-03-31

**Authors:** Jidapa Watcharakitti, Jaturavit Nimnuan, Kuakarun Krusong, Suwat Nanan, Siwaporn Meejoo Smith

**Affiliations:** 1 Center of Sustainable Energy and Green Materials, Faculty of Science, Mahidol University, 999, Phuttamonthon Sai 4 Road, Salaya, Nakhon Pathom 73170, Thailand; 2 Department of Chemistry, Faculty of Science, Mahidol University, 999, Phuttamonthon Sai 4 Road, Salaya, Nakhon Pathom 73170, Thailand; 3 Center of Excellence in Structural and Computational Biology, Department of Biochemistry, Faculty of Science, Chulalongkorn University, 254 Phyathai Rd., Patumwam, Bangkok 10330, Thailand; 4 Department of Chemistry, Faculty of Science, Khon Kaen University, 123 Mittraphap Road, Muang, Khon Kaen 40002, Thailand

**Keywords:** cassava starch, transesterification, K_3_PO_4_, shear strength, paper adhesives

## Abstract

Instead of using finite petroleum-based resources and harmful additives, starch can be used as a biodegradable, low-cost, and non-toxic ingredient for green adhesives. This work employs K_3_PO_4_ catalyzed transesterifications of cassava starch and methyl laurate at varying reaction times (1–10 h), resulting in the enhanced hydrophobicity of starch laurates. At longer reaction times, starch laurates having higher degrees of substitution (DS) were obtained. While starch laurates are the major products of transesterification, relatively low-molecular-weight byproducts (1%) were detected and could be hydrolyzed starches based on gel permeation chromatography results. Contact angle measurements confirmed the relatively high hydrophobicity of the modified starches compared with that of native starch. The modified starches were then employed to prepare water-based adhesives on paper (without any additional additives). Notably, the shear strength of the esterified starch adhesives appears to be independent of the DS of esterified samples, hence the transesterification reaction times. Additionally, the shear strength of water-based adhesives (0.67–0.73 MPa) for bonding to paper substrates is superior to that of two other commercially available glues by a factor of 10 to 80 percent.

## 1. Introduction

**Figure 1:**
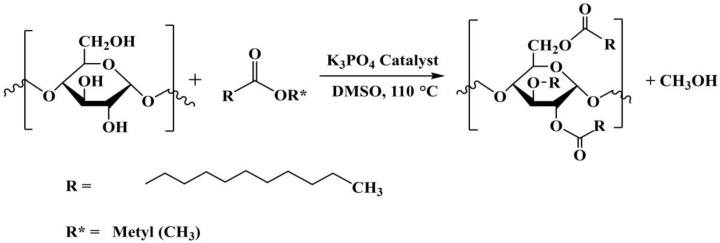
Transesterification of starch and methyl laurate.

Starch is a low-cost, non-toxic, renewable, and biodegradable resource that is widely used in food, medicine, and agriculture. It consists of two biopolymers: amylose and amylopectin. Amylose is made up of long helical chains, while amylopectin has a branched structure [1]. In addition to the applications above, starch is also employed as an ingredient in adhesives for paper and wood [2], especially in the packaging industry, with cassava starch in particular being employed on a large scale for these applications [3,4]. Normally, native starch is not suitable for adhesives due to the presence of many hydrophilic hydroxyl groups in the polymeric structure. These impart poor stability to the adhesive, as manifested by the adhesive not being able to retain its bonding ability to surfaces over time [5]. This issue can be overcome through either the chemical, physical, or enzymatic modification of the starch [6]. In the context of chemical modification, esterification or transesterification, converting the starch hydroxyl groups into more hydrophobic ester groups as shown in Figure 1, can result in improved adhesive properties. The properties of these modified starches are related to the degree of substitution (DS), defined as the average number of hydroxyls replaced by ester groups per glucose unit (typically 0–3). Starches are often reported as either low DS (0.01–0.2), intermediate DS (0.2–1.5), or high DS (1.5–3.0) [7]. Modification of the starch results in changes in water solubility and absorptivity, thermal stability, viscosity, and bonding strength relative to the native material [5], and these directly impact the properties and usefulness of the adhesive. The modifications of starch with olein monomer (vinyl acetate and butyl acrylate) can improve the thermal properties of modified starches, which were further used in wood adhesive application [8]. Maleic anhydride was also employed as a functionalizing reagent to react with starch and to improve the shear strength of wood adhesives [9]. In this research, the use of a dimethyl sulfoxide (DMSO) solvent in transesterification of starch using a base catalyst is a promising method of starch with carboxylic acid ester [10]. Most of the obtained modified starches are used as materials for eco-friendly water-based adhesives for wood [9,11,12], while there have been limited applications for paper. The properties of modified starch and its adhesives were investigated, such as thermal properties, hydrophobicity, structures, and bond strength, using different characterizations.

## 2. Materials and Methods

### 2.1. Materials

Cassava starch (food grade, approximately 82% amylopectin and 18% amylose) was purchased from a local market (Salaya, Nakhon Pathom, Thailand). The used as-received chemicals were potassium phosphate (K_3_PO_4_; Sigma-Aldrich, Munich, Germany), dimethyl sulfoxide (DMSO; AR grade, Central Drug House Ltd., Delhi, India), methyl laurate (Tokyo Chemical Industry, TCI, Tokyo, Japan), chloroform-d (Cambridge Isotope Laboratories, Inc., Cambridge, UK), ethanol, and chloroform (AR grade, RCI Labscan limited, Bangkok, Thailand). Deionized (DI) water was employed as a solvent. 

### 2.2. Method

#### 2.2.1. Cassava Starch Modification

In a general procedure, cassava starch (15 g) was added into a 250 mL round-bottom flask with 150 mL (10% *w*/*w*) of DMSO. The starch was then gelatinized at 90 °C for 3 h before 2% (*w*/*w*) K_3_PO_4_ was added, followed by methyl laurate (1:3 molar ratio of starch: ester). The mixture was then heated at 110 °C for different time periods (1, 2, 4, 6, or 10 h) and on cooling. Then the brownish product was washed with ethanol before being dissolved in chloroform and precipitated by being added to ethanol. Finally, the modified starch was dried at around 80 °C in the oven overnight.

#### 2.2.2. Preparation of Starch-Based Adhesive

Ten grams of cassava (or modified starches) was dissolved in DI water in the weight by volume ratio of 1:20 (starch: water) and heated to 70–90 °C for the gelatinization of each sample. After the starch was gelatinized, the volume of each aqueous starch solution was adjusted to 200 mL and cooled to room temperature.

#### 2.2.3. Characterizations of Starches

##### Degree of Substitution (DS)

Information about the chemical structure and DS value of modified starches was obtained using ^1^H-NMR spectroscopy. Starch laurate samples were dissolved in d6-DMSO and heated at 50 °C until clear solutions were obtained. ^1^H NMR spectra were measured on a Bruker Ascend NMR spectrometer 400 MHz (Billerica, MA, USA) at 23 °C with 32 scans. The DS values of the modified starches were calculated using the following equation [13]:

(1) $$DS=\frac{\left(A\times 7\right)}{\left(3\times C\right)}$$

where *A* is the peak area from the integration from the methyl signals, and *C* is the peak area from the integration of the proton signals from the anhydro glucose unit.

##### Gel Permeation Chromatography (GPC)

Separation of starch components was performed on the principle of size exclusion using GPC, which was performed using an OHpak SB-805 HQ column (6 × 300 mm) with mobile phase 75% (*v*/*v*) DMSO (HPLC grade) at a flow rate of 0.15 mL/min at 60 °C. The retention time was set at 100 min, with the signal being monitored using an RI detector and quantified using LabSolutions (Shimadzu) software. Injection volumes for starch samples were 60 µL after a 10-fold dilution, although that used for standard dextran was 25 µL (10 mg/mL). 

About 4–5 mg of the sample was weighed and heated in 10 mL water at 120 °C for 3 h after cooling down; the solution was filtered by a 0.45 µm nylon syringe filter and followed by a 0.22 µm nylon syringe filter to obtain the final solution for measurement. The sample was filled in a 1.5 mL screw cap vial. The GPC was run as follows: instrument, Agilent Waters 2414 (Santa Clara, CA, USA); column, TSKgel G5000PW (300 × 7.5); system, DI water at a flow rate of 0.5 mL/min with an RI detector; injection volume, 50 µL; column temperature, 80 °C; and detector temperature, 50 °C.

##### Scanning Electron Microscopy (SEM)

The morphology of starch and starch laurate was observed using a JSM-IT500, Jeol, scanning electron microscope (Tokyo, Japan). The samples were placed on a stub with double-sided adhesive tape, and micrographs were recorded at various magnifications.

##### Fourier Transform Infrared (FTIR) Spectroscopy

Modified starch and native starches were analyzed by FTIR spectroscopy on a Nicolet Summit Pro instrument (Thermo Scientific, Waltham, MA, USA) in the attenuated total reflectance (Diamond-ATR) mode using a resolution of 4 cm^−1^ and 64 scans. Spectra were recorded over a region of 4000–650 cm^−1^ [14].

##### Raman Spectroscopy

Raman spectra were collected on a Horiba XploRA PLUS instrument, Japan (combined with LabSpec6 software) equipped with a 785 nm laser providing 28.7 mW of power. The spectra were recorded at a resolution of 5.44 cm^−1^ over a spectral range of 3700 to 50 cm^−1^.

##### Solubility and Swelling Power

The sample (either native cassava starch or starch laurate, 30 mg) was added to an Eppendorf tube followed by 0.6 mL of hot (70 °C) water. After mixing (vortex), the tube was kept in a water bath shaker at 70 °C for 1 h. After this time, the tube was centrifuged at 4000 rpm for 15 min to separate the starch and water phase. The water was removed, and the starch was dried in an oven at 65 °C overnight prior to being weighed [15]. The solubility (%) and swelling power (g/g) of the starch samples were calculated using the following equations:

(2) $$Solubility\;\left(\%\right)=\frac{\left(weight\;of\;starch-weight\;of\;sediment\right)}{weight\;of\;starch}\times 100\%$$

(3) $$Swelling\;Power\;(g/g)=\frac{weight\;of\;sediment\;\left(g\right)}{weight\;of\;\;starch\;\left(g\right)}$$

##### Contact Angle Measurement

The wettability properties of starch powders were estimated by direct observation of the contact angle of a water drop, approximately 5 μL deposited on the surface of the compacted starch powder by using a contact angle meter (SL200KS Model, KINO, Boston, MA, USA). The Young–Laplace equation fitting method was used, and the results were evaluated (shape edge, fitting) by using the Young–Laplace theory [16].

##### Thermal Analysis

Simultaneous thermogravimetric analysis (STA) was carried out on a NETZSCH STA 449 F5 Jupiter instrument (Berlin, Germany) using about 5–10 mg of the sample. The sample was placed in an alumina crucible and heated from 25 to 800 °C with a heating rate of 10 °C/min under nitrogen atmosphere, with a flow rate of 50 mL/min. In addition, differential scanning calorimetry (DSC) was carried out by using a Q200 V24.11 Build 124 TA instrument (New Castle, Delaware, USA). In a typical measurement, approximately 0.5 g of the sample was dispersed in DI water (the ratio 1:4 = sample: water) and stirred for 1 h. A small amount (5 mg) of dispersion was then placed in the pan and heated from 25 to 250 °C (a heating rate of 5 °C/min) under nitrogen atmosphere to observe the gelatinization temperature.

##### Viscosity Measurement

The viscosities of starch-based adhesives were determined in triplicate at 30 °C using a Brookfield viscometer (DV-II) (Ametek, MA, USA) with spindle no.5 at 20 rpm. Values are expressed in mPa·s in this work.

##### Adhesive Bonding Performance

Lap-shear strength, referred to as bonding strength in this study, was obtained for starch-based adhesives following a modification of the ASTM D1002 method. For this, a paper substrate was cut into 100 mm × 25 mm strips. The adhesive (0.04 g) was then applied to a 13 mm × 25 mm region of one paper strip, and then this was overlapped with an identically sized portion of a second paper strip. After the glue dried, the bonding strength was obtained using a universal testing machine (Instron 5965, Norwood, MA, USA) [17].

(4) $$Shear\;strength\;\left(MPa\right)=\frac{Maximum\;loading\;\left(N\right)}{Bonding\;area\;\left(mm^{2}\right)}$$

#### 2.2.4. Statistical Analysis

The results were reported as mean value and standard deviation (SD). Descriptive statistics was calculated using version18 of the predictive analytics software (PASW) program [18]. One-way analysis of variance (ANOVA) was used to test the bonding strength of the starch-based adhesives, followed by a Duncan’s multiple range test to determine significance (*p*-values ≤ 0.05).

## 3. Results and Discussion

### 3.1. Degree of Substitution (DS)

**Figure 2:**
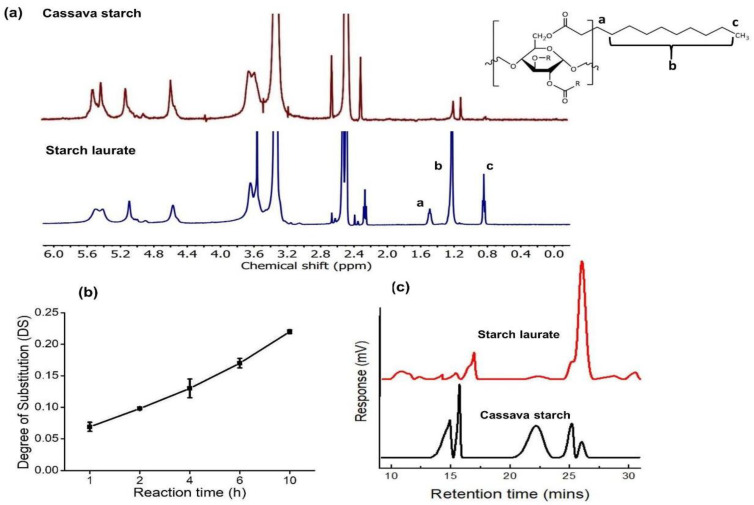
(**a**) ^1^H-NMR spectra of native cassava starch and starch laurate (10 h) while a, b, c denoted the characteristic protons on starch laurate, (**b**) degree of substitution (DS) of starch laurates with the reaction times, and (**c**) aqueous GPC thermograms of cassava starch and starch laurate (10 h).

The values of the degree of substitution were calculated by the mentioned Equation (1). The ^1^H NMR spectra of native cassava starch and starch laurate 10 h in Figure 2a were integrated for the area of the methyl signal (c) at a chemical shift of 0.8 ppm and the peak of four protons from the starch backbone and three hydroxyl groups at 4.4–6 ppm. The DS values of starch laurate products from the reaction of methyl laurate with native starch are in the range of 0.069–0.22 as shown in Figure 2b, and the DS value increases with a higher reaction time.

### 3.2. Gel Permeation Chromatography

Figure 2c shows the GPC chromatograms of cassava starch and starch laurate, with the main peaks of starch observed at retention times of approximately 12–14 min. These peaks were shifted to higher retention in starch laurate, and there is an extra new peak at a retention time of 9 min that indicated that a starch laurate with a higher molecular weight (1.72 × 10^6^ Da) was present. Based on the GPC results, the starch laurates having a higher molecular weight than that of the native cassava starch suggested the achievement of the modification of hydroxyl groups to ester groups. In addition, the GPC peaks around 26 min likely correspond to hydrolytic products resulting from the hydrolysis of polysaccharide by K_3_PO_4_. Transesterification and hydrolysis are in competition with one another. Table 1 contains the details of the molecular weight (M_w_) of all samples, while the reaction times of 4 h and above are sufficient to achieve starch laurates (GPC signal at a retention time of 9 min in water). Furthermore, the results from the DMSO system present that there is an extra new peak in starch laurate samples (29 min) in Table 1 that indicated the hydrolysis product, while the starch laurate peaks were not detected because of its incomplete solubility in DMSO [19,20]. Nevertheless, DMSO may be unsuitable for the detection of esterified starch likely due to the low solubility of starch laurate in DMSO.

### 3.3. Scanning Electron Microscopy (SEM) 

**Figure 3:**
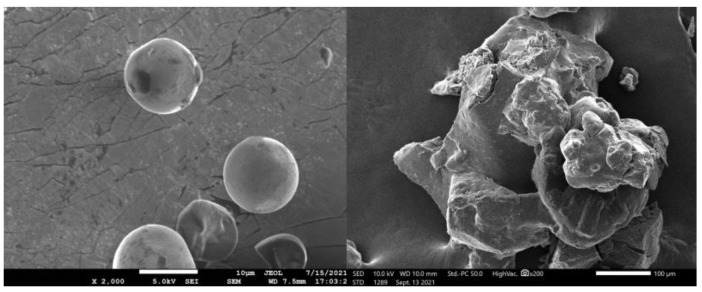
SEM images at 2000× magnification of cassava starch (**left**) and starch laurate, 10 h (**right**).

SEM images (Figure 3) showed the morphology of native and chemically modified starch samples. Native cassava starch (Figure 3, left) displayed spherical granules and a smooth surface, whereas starch laurate (Figure 3, right) exhibited an irregular-shaped, rough surface of starch granule aggregations [21] due to the intermolecular and intramolecular hydrogen bond breakdown. 

### 3.4. Fourier Transform Infrared (FTIR) Spectroscopy

**Figure 4:**
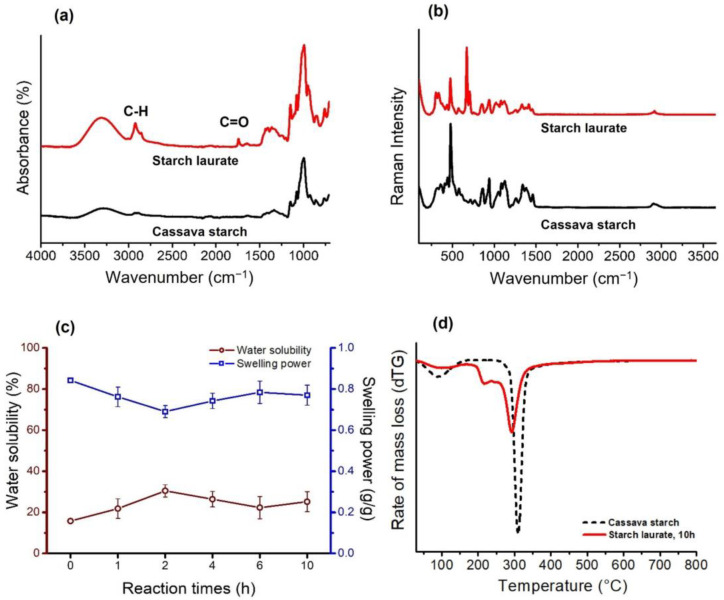
(**a**) FTIR spectra of cassava starch and starch laurate (10 h). (**b**) Raman spectra of cassava starch and starch laurate (10 h). (**c**) Water solubility (%) in red line and Swelling power (g/g) in blue line of native cassava starch (0 h) and starch laurate with different reaction times. (**d**) Derivative thermal gravity (dTG) thermogram cassava starch and starch laurate (10 h).

Figure 4a illustrates the FTIR spectra of the starch laurate samples and native starch. The presence of carbonyl stretching vibrations (1740–1750 cm^−1^) in all modified starch confirmed the successful transesterification of the starch. In addition, the C–H stretching vibrations corresponding to the alkyl groups from the laurate fatty acid ester chain are present at around 2920 and 2850 cm^−1^. The broad band in the region of 3000–3600 cm^−1^ is attributed to hydroxyl vibration and decreased in modified starch samples, suggesting the conversion of hydroxyl groups to ester groups. Characteristic peaks (C–O stretching) from the starch polysaccharide backbone are observable at 900–1250 cm^−1^ in all samples [5,22].

### 3.5. Raman Spectroscopy

Raman spectra in Figure 4b show the presence of many bands between 440 and 675 cm^−1^, which correspond to the skeletal vibrations of the pyranose ring in the starch glucose units. A peak between 920 and 960 cm^−1^, a result of vibrations in the C–O–C or the 1,4 glycosidic linkages [14], occurs in all starch samples but is of higher intensity in the native material. In contrast, C–OH bending, as evident in all starch samples between 1085 and 1130 cm^−1^, is of lower intensity in starch laurates. A peak at approximately 2920 cm^−1^, diagnostic of C–H stretching, can be observed in all starch samples [23].

### 3.6. Water Solubility and Swelling Power of Starches

Figure 4c indicated that the swelling power of starch laurate was lower than that of native starch, while the solubility increased from the value of the native. The swelling power indicates the uptake of water during the gelatinization; hot water is adsorbed in the molecules of starch granules and caused them to swell [24]. The solubility and swelling power were associated with the linkage strength within the starch structure. The cleavage of the interaction between the glycosidic linkages of starch can occur from transesterification, so the hydrogen bond from the granular starch is prevented to bind with the surrounding hydroxyl group of water, which results in the lower swelling power of the modified starch [15]. Moreover, the intermolecular hydrogen bond between the starch chains was disrupted by the substitution group, thereby decreasing the entanglement of the starch laurate chains that could have caused the increasing solubility in water [25]. These results indicate that the esterified starch obtained from methyl laurate reactions is suitable for water-based adhesive preparation.

### 3.7. Thermal Properties of Starches

Figure 4d displays the derivative thermograms (dTG) of native cassava starch and starch laurate (MeL, 10 h). At approximately 100 °C, a mass loss (9.51%) from the native starch sample was due to water evaporation. Consistent with the increased hydrophobicity of starch laurate, starch laurate samples evaporated less water [26]. The decomposition temperature of starch laurates falls within the range of 216–220 °C, as shown in Table 2. Based on Figure 4d, the decomposition of starch laurates began at around 220 °C, which is lower than that of native cassava starch (dash curve). This could be the degradation of the main polymer, and another peak in starch laurate was likely derived from the hydroxyl groups of the unmodified portions of starch. The results indicated a decrease in the thermal stability of starch laurates compared with native starch, in agreement with the 2013 report by Lu et al. In contrast, modification raises the gelatinization temperature in Table 2 as a result of the increased distance between the molecules at the surface, which affects the modified starch’s interaction with water [27].

### 3.8. Contact Angle

**Figure 5:**
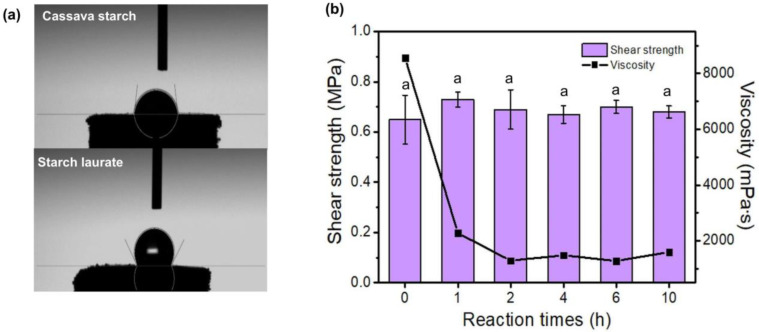
(**a**) Contact angle images of cassava starch and starch laurate (10 h) and (**b**) shear strength and viscosity of cassava starch adhesive and starch laurate adhesives. Group classification (a) was given to the data based on the statistical analysis results (significant difference; *p* ≤ 0.05).

The hydrophobicity of starches was determined by measuring the contact angle of water on the pelletized samples. An angle lower than 90° is considered hydrophilic, between 90° and 120° is considered hydrophobic, and materials exhibiting angles greater than 120° are termed superhydrophobic [28]. Due to the grafting of long-chain ester functionalities to the starch backbone, starch laurates are more hydrophobic (contact angle = 88.75°–132.36°) than the native cassava starch (contact angle = 82.16°). Table 2 indicates the water resistance of starch laurates because of the presence of laurate on the starch structure. Representative images of water droplets on the surface of native starch and starch laurate are shown in Figure 5a and Figure S1.

### 3.9. Viscosity and Bond Strength

Due to their solubility in water and ability to swell, starch laurate samples were utilized to prepare water-based adhesives. The gelatinized aqueous starch laurate samples showed moderately low viscosity, which ranged from 1280 to 2280 mPa·s (Figure 5b). On the other hand, the viscosity of native starch adhesive is quite high, at 8553 mPa·s; this may be due to the propensity for hydrogen bonding interactions, which help adhesives to spread and adhere to surfaces [29]. As a result of the transesterification process, the short-chain starch byproduct or hydrolyzed starches [30] may act to reduce order in the arrangement of starch chains in water or make the polymeric chains more flexible [31]. The significantly decreased viscosity (approximately 73% lower) may be due to the hydrolytic cleavage of the original starch chain upon transesterification (GPC results, Table 1). In an excellent agreement with this work, the viscosity of adhesives made from acid hydrolyzed potato starch [4] was reported to be lower than that of native starch. Notably, the lower viscosity of starch laurate adhesives might improve the glue spreadability, promoting interface interactions between the glue and paper substrates [11].

Shear strength, which represents the ability of adhesives to bond surfaces together, is a significant factor in determining the usefulness of such products. In a previous work, paper glues were made from whey protein, and various additives show quite high shear strengths (1.07–1.35 MPa) [17,32]. In this study, starch laurate adhesives exhibit shear strengths in the range of 0.67–0.73 MPa (Figure 5b), which are comparable with those of native-starch-based adhesives (insignificantly different, *p* > 0.05). The failure mode after the shear strength measurement indicated cohesive failure in paper substrates due to the higher bonding strength between the adhesive and adherend than their individual strength [33]. Hydrogen bonding interactions between the hydroxyl groups in starches and cellulose in papers are the main contribution of the interfacial adhesion [34]. From the results in Figure 5b, no negligible shear strengths were observed for all adhesive samples, suggesting that the laurate group on starch esters gave no significant medication of hydrogen bonds between the cellulose and modified starch. Nonetheless, the shear strength of all starch adhesives was greater than that of two commercially available water-based glues with their shear strengths ranging from 0.41 to 0.58 MPa. As a result, starch esters derived from cassava starch are suitable raw materials to produce water-based adhesives for paper applications. 

### 3.10. Effect of Reaction Times on the Properties of Starch Laurates

According to Table 2, the thermal properties (gelatinization and decomposition temperatures) and water wettability of native starch and starch laurates are significantly different. The higher contact angles and gelatinization temperatures of starch laurates, compared with that of the native starch, are expected as the starch laurate granules have lower water susceptibility due to the decreased hydrogen bonding interactions between water and the hydrophobic starch. These results agree well with a previous work [27]. Nonetheless, there is no evident correlation between the DS of starch laurates formed at varying reaction times and their thermal properties. Possible reasons are described as follows. Due to the moderately low reactivity of methyl laurate, starch transesterification reactions gave starch laurate products with low DS values (<0.25) [7]. Based on the GPC results in Table 1, the comparable molecular weights of starch laurates formed at varying reaction times may suggest quite similar hydrogen bonding interactions in an aqueous environment. As a result, it is likely that the gelatinization temperature of starch laurate samples (94–97 °C), as well as their decomposition temperature (216–222 °C), is quite the same. From Table 2, this work suggested that the hydrophobic starch laurate samples can be obtained by increasing the transesterification reaction times (≥2 h). A contact angle above 90° indicates hydrophobicity [35]. The water wettability of starch laurate granules is greatly influenced by their DS as a result of the long chain of laurate attached on the starch backbone. On the other hand, the effects of the DS of starch laurate materials on the shear strength of adhesives were negligible, implying that the bonding strength of the water glues on papers was not compromised by the laurate groups functionalized on the starch. 

## 4. Conclusions

Starch laurates, produced via a K_3_PO_4_ catalyzed transesterification of cassava starch in the presence of methyl laurate, were employed to prepare water-based adhesives to bond papers. A low DS of 0.22, from a maximum possible DS of 3.0, was obtained. Aqueous GPC analyses confirmed the successful modification of cassava starch. The enhanced hydrophobicity of starch laurates suggested the improved water resistance of modified starch adhesives. The starch laurate water-based glues exhibit superior adhesion strength for paper compared with commercially available paper glues. Thus, the reactivity of methyl laurate, which was determined in this study, and the enhanced hydrophobicity of starch laurates can lead to the development of environmentally friendly adhesives with superior water resistance and adhesion strength for paper. Further investigation on the effects of humidity on the adhesives’ bonding strength can be carried out to add more confidence that hydrophobic starches (e.g., starch laurates) are suitable raw materials for the production of water-based adhesives with enhanced water resistance compared with native starches.

## Figures and Tables

**Table 1:** Molecular weight (M_w_) of cassava starch and starch laurates in water and DMSO solvents (n.d. = not detected).

Rx. Time (h)	Water			DMSO	
	M_w, t = 9 min_ (Da)	M_w, t = 12–14 min_ (Da)	M_w, t = 26 min_ (Da)	M_w, t = 0.7 min_ (Da)	M_w, t = 29 min_ (Da)
0	n.d.	5.58 × 10^5^	8.02 × 10^3^	4.80 × 10^11^	n.d.
1	n.d.	4.08 × 10^5^	7.95 × 10^3^	4.79 × 10^11^	1.87 × 10^8^
2	n.d.	3.76 × 10^5^	7.98 × 10^3^	4.82 × 10^11^	1.80 × 10^8^
4	1.47 × 10^6^	3.80 × 10^5^	8.06 × 10^3^	4.81 × 10^11^	1.94 × 10^8^
6	1.63 × 10^6^	4.45 × 10^5^	8.26 × 10^3^	4.81 × 10^11^	1.94 × 10^8^
10	1.72 × 10^6^	3.28 × 10^5^	8.50 × 10^3^	n.d.	1.92 × 10^8^

**Table 2:** Thermal properties of cassava starch and starch laurates.

	Rx. Time (h)	DS ^NMR^	Gelatinization		Decomposition Temp. (°C)	Contact Angle (Degree, °)
			Temp. (°C)	ΔH (J/g)		
Native starch	0	0	65.8	1.13	290.4	82.2
Starch laurate	1	0.07	96.9	6.06	216.8	88.7
	2	0.10	94.6	50.3	218.0	97.9
	4	0.13	95.9	31.8	218.9	120.5
	6	0.17	94.7	19.3	220.9	127.3
	10	0.22	95.2	9.82	222.4	132.4

## Data Availability

Data are available upon request.

## Supplementary Materials

The following supporting information can be downloaded at: https://www.mdpi.com/article/10.3390/polym15071754/s1, Figure S1: Contact angle images of cassava starch and starch laurate.
