# Supplementary material for: Insight into the Molecular Weight of Hydrophobic Starch Laurate-Based Adhesives for Paper
Source: Polymers (Basel). 2023 Mar 31;15(7):1754. doi: 10.3390/polym15071754 (PMC10097198; doi:10.3390/polym15071754)
Supplement: Supplementary file 1 [file polymers-15-01754-s001.zip › polymers-2275048-supplementary.pdf]

Insight into the Molecular Weight of Hydrophobic Starch Laurate-Based Adhesives for Paper

Jidapa Watcharakitti [1,2], Jaturavit Nimnuan[1,2], Kuakarun Krusong [3], Suwat Nanan [4] and Siwaporn Meejoo Smith [1,2,*]

[1] Center of Sustainable Energy and Green Materials, Faculty of Science, Mahidol University, 999, Phuttamonthon Sai 4 Road, Salaya, Nakhon Pathom 73170, Thailand
[2] Department of Chemistry, Faculty of Science, Mahidol University, 999, Phuttamonthon Sai 4 Road, Salaya, Nakhon Pathom 73170, Thailand
[3] Center of Excellence in Structural and Computational Biology, Department of Biochemistry, Faculty of Science, Chulalongkorn University, 254 Phyathai Rd., Patumwam, Bangkok, 10330, Thailand
[4] Department of Chemistry, Faculty of Science, Khon Kaen University, 123 Mittraphap Road, Muang, Khon Kaen 40002, Thailand
* Correspondence: siwaporn.smi@mahidol.edu; Tel.: +66-(0)93-5939449

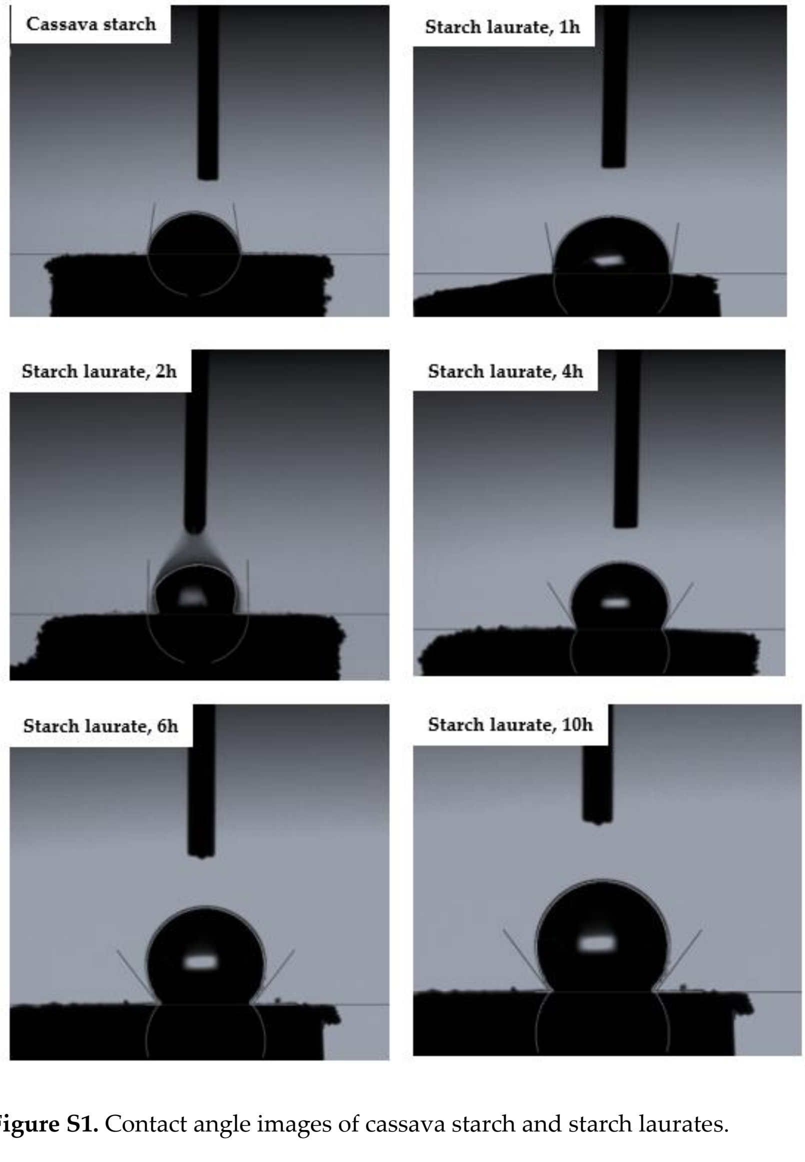

Figure S1. Contact angle images of cassava starch and starch laurates.
